# Differential Expression of *AhR* in Peripheral Mononuclear Cells in Response to Exposure to Polycyclic Aromatic Hydrocarbons in Mexican Women

**DOI:** 10.3390/toxics11010028

**Published:** 2022-12-28

**Authors:** José Antonio Varela-Silva, Miguel Ernesto Martínez-Leija, Sandra Teresa Orta-García, Ivan Nelinho Pérez-Maldonado, Jesús Adrián López, Hiram Hernández-López, Roberto González-Amaro, Emma S. Calderón-Aranda, Diana Patricia Portales-Pérez, Mariana Salgado-Bustamante

**Affiliations:** 1 Biochemistry Department, and Immunology, Department of Faculty of Medicine, UASLP, San Luis Potosí 78000, Mexico; 2 Laboratory of Molecular Toxicology, Centro de Investigación Aplicada en, Ambiente y Salud (CIAAS), Coordinación para la Innovación y Aplicación de la, Ciencia y la Tecnología (CIACYT), UASLP, San Luis Potosí 78000, Mexico; 3 Laboratorio de microRNAs y Cáncer, Unidad Académica de Ciencias Biológicas, Universidad Autónoma de Zacatecas, Zacatecas 98066, Mexico; 4 Academic Unit of Chemistry Sciences, Universidad Autónoma de Zacatecas, Zacatecas 98606, Mexico; 5 Laboratory of Immunology Cellular and molecular, Faculty of Chemistry, Universidad Autónoma de San Luis Potosí, San Luis Potosí 78000, Mexico; 6 Toxicology Department, Centro de Investigación y Estudios Avanzados, IPN, México City 07360, Mexico

**Keywords:** pollutants, women exposure, microRNAs, PAHs

## Abstract

The exposure to air pollutants causes significant damage to health, and inefficient cooking and heating practices produce high levels of household air pollution, including a wide range of health-damaging pollutants such as fine particles, carbon monoxide and PAHs. The exposure to PAHs has been associated with the development of neoplastic processes, asthma, genotoxicity, altered neurodevelopment and inflammation. The effects on the induction of proinflammatory cytokines are attributed to the activation of *AhR*. However, the molecular mechanisms by which the PAHs produce proinflammatory effects are unknown. This study was performed on a group of 41 Mexican women from two rural communities who had stoves inside their houses, used wood as biomass fuel, and, thus, were vulnerable. According to the urinary 1-OHP concentration, the samples were stratified into two groups for determination of the levels of *TNF-α*, *AhR*, *CYP1B1*, miR-125b and miR-155 expression. Our results showed that the *CYP1B1*, *TNF-α*, miR-125b and miR-155 expression levels were not statistically different between women with the lowest and highest levels of 1-OHP. Interestingly, high levels of PAHs promoted augmented expression of AhR, which is a protein involved in the modulation of inflammatory pathways in vivo, suggesting that cell signaling of *AhR* may be implicated in several pathogenesis processes.

## 1. Introduction

Environmental pollutants are generated as a result of human activity; around 3 billion people still cook and heat their homes using solid fuels (i.e., wood, crop wastes, charcoal, coal and dung) in open fires and leaky stoves, and 4.3 million people a year die prematurely from illnesses attributable to household air pollution caused by the inefficient use of solid fuels [1,2]. Some compounds such as polycyclic aromatic hydrocarbons (PAHs) are generated by the incomplete combustion of organic matter and can travel together with coarse (10 PM), fine (2.5 PM) and ultrafine particles (0.1 PM) across long distances, being deposited and accumulating in various environmental matrices and entering into living organisms, bioaccumulating and becoming bioavailable to higher organisms such as humans. Additionally, contact with PAHs causes a biomagnification as they enter the food chain [1,2,3,4].

Benzo [a] pyreno (BaP) is one of the PAHs most widely studied for its effects on health, including inflammatory diseases and cancer. The majority of its effects have been associated with the activation of AhR, which is a factor with bHLH domains (basic helix–loop–helix) [5]. AhR is a ligand-dependent transcription factor that is stripped from Hsp90, p23 and KAP2 proteins when activated and dimerizes with AhR nuclear translocator (ARNT)-recognizing DNA regulatory elements called XREs or DREs (xenobiotic response elements or dioxin response elements) of target genes, thereby increasing their transcription. The isoforms of Cytochromes p450 (CYP1A1, CYP1A2, CYP1B1) are targets of AhR that participate in PAH metabolism. CYP1A1 and CYP1B1 promote the transformation of B[a]P to B[a]P-7,8-oxide through a hydration reaction by the enzyme epoxide hydrolase B[a]P-7,8-oxide, and it is subsequently metabolized to B[a]P-trans-7,8-dihydrodiol (B [a] P-7,8-DHD). B [a] P-7,8-DHD is a substrate of CYPB in a second oxidation reaction generating the final carcinogenic metabolite [a] P-7,8-dihydroxy-9,10-epoxide (BPDE). BPDE joins the DNA chain, principally generating deoxyguanosine adducts [6,7].

Currently, most of the effects of BaP are attributed to the activation of AhR, which regulates the transcription of genes controlled by DRE sequences that have been found in promoter regions of different cytokines in murine models, including IL-2, IL-5, IL-10, TGF-β1 and IFNɣ [8]. However, it has been found that the expression of proinflammatory genes without DRE sequences in their promoters is upregulated with exposure to BaP, suggesting that there is another type of independent regulation of AhR through the regulation of protein and non-coding RNAs.

Epigenetic modification is one of the mechanisms by which gene expression is regulated, and it is characterized by the presence of changes in the composition and structure of chromatin, which can potentially be inherited [9]. Three of the most significant epigenetic modifications are DNA methylation, acetylation and expression of microRNA (miRNA). miRNAs are 18 to 22 nucleotides in length and regulate gene expression at the protein-translation and messenger RNA (mRNA)-level in P bodies by mRNA sequestration that results in a negative correlation between miRNA and mRNA. miRNA–mRNA interactions can explain the increase or decrease in protein levels [10]. Currently, in vitro models have demonstrated the involvement of microRNAs in the biological responses induced by toxic compounds such as BaP; therefore, altered expression of miRNAs is considered a biomarker [11]. In the present work, we analyzed the induction of the proinflammatory cytokine *TNF-α* attributed to the activation of *AhR* and *CYP1B1* as metabolic processors of PAH, and miR-125b and miR-155 expression related to these processes. We found that *AhR* expression is related to PAH exposure, which may be implicated in several pathogenesis processes.

## 2. Materials and Methods

### 2.1. Population

Forty-one samples were analyzed from healthy women with an age range of 18–45 years old, and all were residents of two rural communities (with little vehicular traffic) in the state of San Luis Potosi, Mexico (The Cañon, in the municipality of Xilitla, and Comoca Ahuacatitla, in the municipality of Axtla Terrazas). The women who participated in the study used wood as their only source of fuel for cooking, lived in traditional houses (usually wood) and usually spent most of the day inside their homes and an average of 6.5 h cooking. All the women who participated in the study had lived in their community since they were born. Therefore, their main source of exposure to PAHs was the combustion of biomass. After informed consent was obtained, a questionnaire was completed and urine samples were taken. The questionnaire included characteristics such as age, weight, height and exposure to snuff smoke. In addition, sociodemographic characteristics were described. The study was approved by the ethics committee of the Faculty of Medicine at the Autonomous University of San Luis Potosi.

### 2.2. Urine Collection

The first morning urine of each woman (at approximately 7:00 am) was collected. The urine was collected in airtight plastic bottles and stored in a freezer at −20 °C until analyzed. Before analysis, samples were thawed at room temperature, homogenized, and 10 ml of urine was transferred to a test tube (Corning®, New York, NY, USA).

### 2.3. Determination of Urinary 1-OHP

1-OHP (half-life ranged from 6 to 30 h) was taken as a representative biomarker of exposure to PAH mixtures [12,13]; it was taken into account that this compound is a pyrene metabolite, and, in turn, pyrene is often present in PAH mixtures. 1-OHP was quantified following the method described previously [13,14]. The analyses were performed using HPLC (HP1100, Agilent Technologies; Santa Clara, CA, USA) and a fluorescence detector (G1321A). A Zorbax SB-C18 pre-column (Agilent Technologies; Santa Clara, CA, USA) and a Zorbax Eclipse XDB-C18 column (Agilent Technologies; Santa Clara, CA, USA) were used. The analysis temperature was set at 40 °C, the flow was adjusted to 1 mL/min and the injection volume was 20 μL. 1-OHP was eluted with 88:12 methanol:water and 1% ascorbic acid. Data were collected and processed using HP ChemStation software (Dayton, OH). Urinary 1-OHP concentrations were adjusted to urinary creatinine, which was determined using the Jaffe colorimetric method [15]. Under our conditions, the detection and quantification limits were 1.0 nmol/L and 3.0 nmol/L, respectively. Quality control was certified using the standards IRIS Clin Cal Recipe (Munich, Germany) 50013, 8867 and 50014 (9.1, 15.6 and 32.5 nmol/L 1-OHP, respectively), and there was a recovery rate of 99%. 

### 2.4. Peripheral Blood Mononuclear Cells (PBMCs) Isolation

Venous blood was collected by venipuncture from the antecubital area of the arm into tubes (Becton Dickinson Vacutainer® Mexico) containing EDTA. Peripheral blood mononuclear cells (PBMCs ) were separated by density gradient centrifugation using Ficoll-Hypaque (Sigma-Aldrich by Merck, Darmstadt, Germany), and washed with phosphate-buffered saline (PBS) solution (Sigma-Aldrich by Merck, Darmstadt, Germany). 

### 2.5. RNA Isolation and RT-qPCR

The PBMCs were collected and washed with PBS for total RNA and miRNA isolation using Trizol (Invitrogen by Thermo Fisher Scientific, Waltham, MA, USA) method; the concentration and quality was determined by spectrophotometric analysis in a Synergy HT Multi-Mode Microplate Reader, using Gen5™ Data Analysis Software (BioTek Instruments, Inc., a part of Agilent, Santa Clara, CA, USA), and the samples were stored at −80 °C until use. Complementary cDNA was synthesized with 1 μg total RNA using reverse transcriptase superscript II and reverse transcription reagents of Invitrogen (Thermo Fisher Scientific, Waltham, MA, USA) under the following conditions: 25 °C 10 min, 35 °C 90 min, 94 °C 5 min and 4 °C 5 min. The qPCR was performed by mixing 1 μL of cDNA (100 ng/μL), 0.1 μL sense and antisense primers (20 pM) (Invitrogen Thermo Fisher Scientific, Waltham, NA, USA) and 1X SYBR® Green PCR Master Mix (*Applied Biosystems* by Life Technologies Waltham, MA, USA) in a total volume of 10 μL. Primers were designed and checked for specificity by BLAST search, and the purity of the PCR products and specificity of the reaction were checked by agarose gel electrophoresis analysis. The expression of target genes was determined using the CFX96 BioRad thermocycler (Bio-Rad Laboratories Hercules, CA, USA). The cycling conditions were as follows: 10 min denaturing at 95 °C, followed by 40 cycles of denaturing at 95 °C for 15 s, 45 s primer annealing, and elongation at 60 °C for 18 s; specifically, the annealing temperatures were 67.5 °C for *TNFα* and *AhR* and 66.5 °C for *CYP1B1*. The melting curve was analyzed with CFX Manager™ software (Bio-Rad Laboratories Hercules, CA, USA). Transcript expressions were normalized to *18S* rRNA housekeeping gene and data were quantified by the method of 2^−ΔΔCt^. The primers used were as follows:
GenesForward sequence (5′-3′)Reverse sequence (5′-3′)Weight of the ampliconTm °CNumber of Cycles18sCGGCTACCACATCCAAGGAAGCTGGAATTACCGCGGCT189 pb6040*TNFα*CCCACGGCTCCACCCTCTCTTCTGGGGGCCGATCACTCCA215 pb67.540*AhR*TCATTTGCTGGAGGTCACCCGCCAAGGACTGTTGCTGTTG254 pb 6040*CyP1B1*TAGTGGTGCTGAATGGCGAG CTCCGAGTAGTGGCCGAAAG137 pb66.540

### 2.6. miRNA Expression 

For microRNA analysis, reverse transcription (RT) and real-time quantitative polymerase chain reaction (qRT-PCR) were performed using a TaqMan® MicroRNA Assay (Applied Biosystems by Life Technologies, Waltham, MA, USA; miR-155: 002623, miR125b:000449) according to the instructions supplied by the manufacturer. Small nuclear RNA U6 was used for normalization.

### 2.7. Statistical Analysis

Data are presented as mean ± SEM values. An unpaired *t*-test was performed to identify changes in the gene expression of *AhR*, *CYP1A1*, *TNF-α*, hsamiR-125 and hsamiR-155, and a Pearson coefficient test was applied to determine gene expression correlations. All statistical analysis was performed with the Graph Pad Prism version 8.0 for Windows (Prism Software, La Jolla, CA, USA, www.graphpad.com). A *p* value less than 0.05 was considered significant.

## 3. Results

### 3.1. 1-OHP Determination in Urine Samples

**Figure 1:**
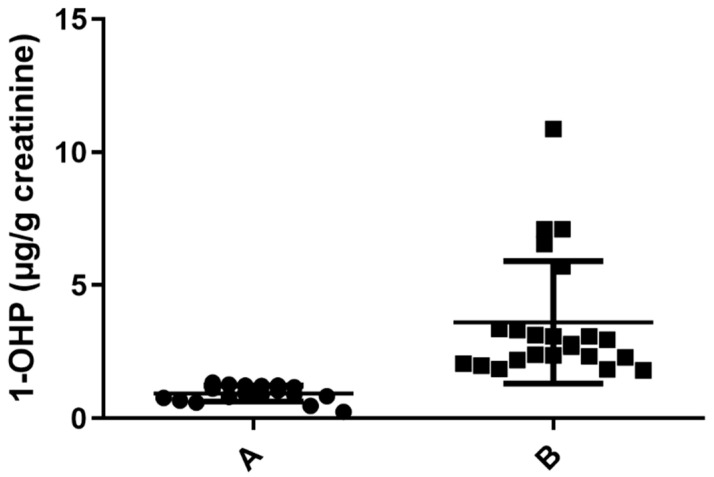
Determination of 1 -OHP in µg/g creatinine in urine samples exposed to PAHs. Samples with urine concentration of 1-OHP ≤ 1.46 µg/g creatinine (*n* = 18) A. Samples with concentration of 1-OHP > 1.46 µg/g creatinine (*n* = 23), (*p* = 0.0001) B.

PAHs are generated during the incomplete combustion of organic matter. To determine the level of exposure to PAHs, we evaluated the concentration of urinary 1-OHP as a biomarker of exposure to the hydrocarbon mixture, as previously shown [16,17]. We assessed 41 urine samples of healthy women who had a traditional stove inside their house and used wood as a biomass fuel for cooking. Normalized data and non-normalized data with urine creatinine concentration (geometric mean ± SD 1.46 ± 2.11 μg/g creatinine; 1.09 ± 1.68 μg/L) and percentiles are shown in Table 1. Interestingly, in PC25, a value of 0.84 μg 1-OHP/g creatinine was found, despite limit values of 0.463 μg 1-OHP/g creatinine for occupationally unexposed non-smoking individuals and 1.46 μg 1-OHP/g creatinine for the occupationally unexposed smoking population having been previously established [13]. Considering that the women included in our study were occupationally unexposed but were in contact with a source of pollutants due to incomplete combustion, we stratified the population into two groups: group A, including individuals with less exposure to PAHs (levels below 1.46 μg 1-OHP/g creatinine), and group B, including individuals with greater exposure (levels above 1.46 μg 1-OHP/g creatinine) (Figure 1). The collected biochemical and anthropometric data from the studied population showed statistical difference solely for 1-OHP by the multiple *t*-tests, as seen in Table 2. Other features of the population, such as age, glucose level, cholesterol, height, weight, etc., were not statistically significant, reaffirming that 1-OHP could be used to stratify people exposed to PAHs. 

### 3.2. Determination and Correlation of AhR, CYP1B1 and TNF-α Expression by RT-qPCR

**Figure 2:**
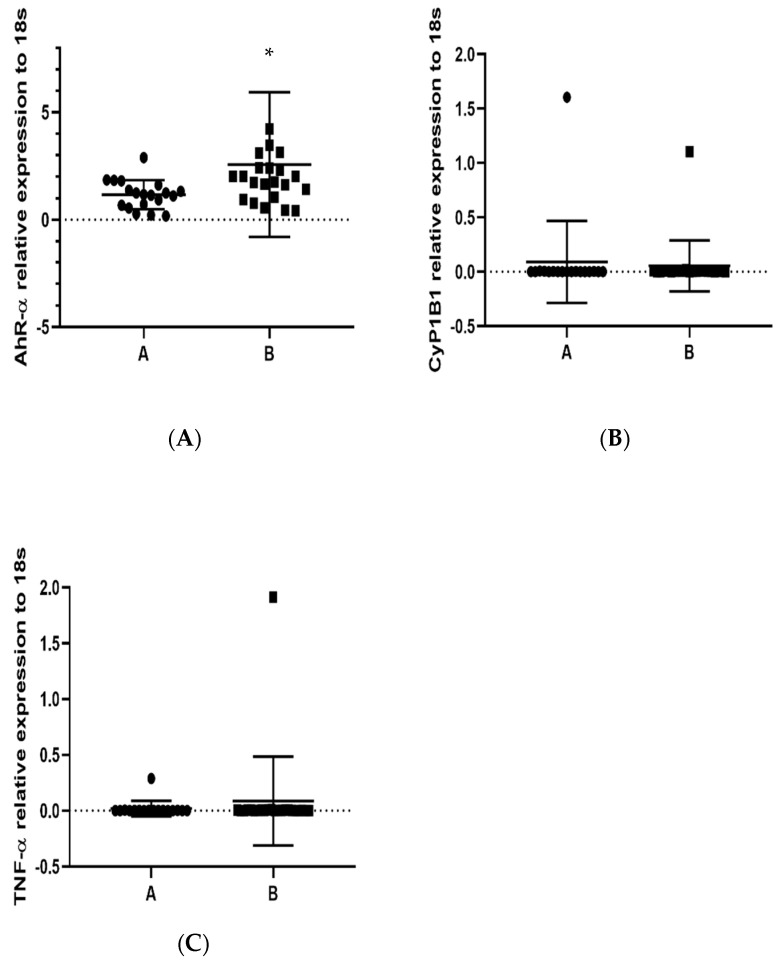
Determination by RT-qPCR of *AhR*, *CYP1B1* and *TNF-α* relative expression in PBMCs in population (groups A and B) exposed to PAHs: (**A**) *AhR*, * *p* = 0.0412; (**B**) *CYP1B1*, *p* = 0.3570; and (**C**) *TNF-α*, *p* = 0.2476. 18s RNA was used to normalize the expressed transcripts. * *p* < 0.05.

Once the 1-OHP concentration was determined and groups A and B were assigned as low and high 1-OHP concentration, respectively, we used the lower exposure group (“A”) as a calibrator for the group with the highest exposure (“B”). We analyzed the expression levels of *TNF-α*, *AhR* and *CYP1B1* mRNAs in groups A and B. The difference in *AhR* expression between groups A and B was statistically significant, *p* = 0.0412; in group B, an increase was observed (Figure 2A). The expression of *CYP1B1* recorded similar expression in both groups, *p* = 0.3570 (Figure 2B), and *TNF-α* expression showed even more similarity between groups than *CYP1B1* (Figure 2C), *p* = 0.2476. Exposure to PAHs augmented *AhR* expression, suggesting a common gene regulation in response to contact with PAHs. 

### 3.3. Evaluation of miR-125b and miR-155 Relative Expression in a Population Exposed to PAHs

**Figure 3:**
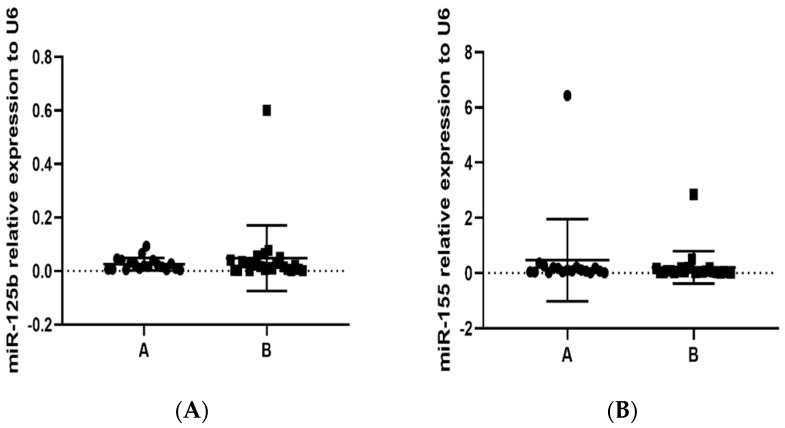
Determination by RT-qPCR of miR-125b and miR-155 relative expression in PBMCs in population (groups A and B) exposed to PAHs: (**A**) miR-125b, *p* = 0.2201; and (**B**) miR-155, *p* = 0.2227. Small nuclear RNA U6 was used for normalization.

It is well known that the regulation of gene expression is complex; currently, epigenetic regulators may explain part of the control. In this context, we evaluated the expression of miR-125b, an important master regulator of the methylation process, and miR-155, considered a pro-inflammatory regulator that is implicated in several pathologies. We did not find statistically significant differences in the expression of miR-125b and miR-155 between study groups (Figure 3A,B). 

## 4. Discussion

Women who use biomass as the main source of energy for cooking their food and heating their homes present different biomarkers of pollutants in their bodies such as 1-OHP. They are directly and chronically exposed to pollutants generated during their daily activities and spend a significant amount of time inside their kitchens every day. In this work, all of the samples tested presented values of 1-OHP higher than 0.463 μg/g creatinine, a limit value proposed for a non-occupationally exposed, non-smoking population [13]. We obtained levels almost three times higher (1.09 ± 1.68 μg/L) than Polanska et al., 2014, who reported 0.43 μg 1-OHP/g creatinine [18]. Furthermore, in 2016, Pruneda-Álvarez et al. reported a value of 0.92 ± 0.92 in a study performed in Mexican indigenous and rural communities [19]. Interestingly, in another study in the rural community El Leoncito, San Luis Potosí, the mean value of 1-OHP was 0.56 μg/L for 40 women who cooked on rustic stoves in their homes [20]; the value was similar to the 0.5 μg/L that was established in a German environmental survey for a general population without risk [21]. In both studies, Pruneda and Ruiz included indigenous Mexican women; however, the values were half of those found in the current study. The National Health and Nutrition Examination Survey IV (NHANES IV) reported a geometric mean of 0.074 μg/L for 1-OHP in people aged 20 years and older (*n* = 1301), a value ten times lower than our results; however, whether they are non-smokers and whether or not they are exposed to either source of PAHs were not specified [22]. Additionally, it should be considered that location, type of house, gender, age and genetic differences could all contribute to 1-OHP concentrations. Pruneda-Álvarez et al., 2012, showed that the levels of 1-OHP in indigenous women exposed either outdoors or indoors were 0.73 ± 0.45 μg/L and 4.81 ± 9.6 μg/L, respectively; the highest levels were in women who had a traditional stove inside their home and spent around 8 h cooking daily [23]. The exposure scenario is similar to our study; however, the mean for 1-OHP μg/L in the Pruneda-Álvarez 2012 report was significantly higher than what is reported here. Exposure to a mixture of PAHs could be an important human health risk factor, and this exposure has been associated with several adverse health effects [22]. The molecular mechanisms related to the health effects include DNA adducts, increased apoptosis, oxidative damage and pro-inflammatory responses [24,25,26,27]. In this regard, we evaluated mRNA levels of *TNF-α* as a pro-inflammatory biomarker in the women’s samples and our results indicated slight upregulation of mRNA in the high-exposure group, suggesting a probable pro-inflammatory response to PAH exposure. In a recent study performed on 39 taxi drivers potentially exposed to emissions, a positive linear correlation between 1-OHP levels and pro-inflammatory cytokines (IL 1β (*r* = 0.37, *p* = 0.007), IL-6 (*r* = 0.32, *p* = 0.02) and TNFα (*r* = 0.33, *p* = 0.02)) was reported [28], suggesting a weak correlation between PAH exposure and TNFα mRNA and protein expression. The PAHs regulated gene expression at several levels, cell systems and cell-signaling pathways. It was shown that *p,p*′-Dichlorodiphenyldichloroethylene (DDE) and coplanar 3,3′,4,4′,5-pentachlorobiphenyl (PCBs) compounds were able to enhance *AhR* transcript expression [24,25]. Recently, enhanced AhR expression has been related to an inflammatory state as a consequence of 2,3,7,8-tetrachlorodibenzo-*p*-dioxin (TCDD), PCBs, DDE and metabolites exposure [6,26,29,30]. Here, we found an augmented expression of *AhR* in the high-exposure group, suggesting that PAHs promote responses through AhR activation. It has been shown that an increase in TNF-α expression contributes to keeping the activation of the AhR pathway and the chronic inflammatory state producing a positive feedback. AhR is a key regulatory element for some xenobiotic degradation enzymes, notably cytochromes P450 belonging to the CYP1 family. Cytochrome activation is induced by several ligands of AhR, such as TCDD or PAHs such as BaP, as potent inductors of CYP1B1 that could be considered as a biomarker for the activation of AhR [27]. AhR is mainly expressed in liver cells, but it is also present in different types of cells, such as blood cells [29,30,31], suggesting that it could be used as a biomarker for PAH exposure. 

Recently, epigenetic mechanisms have emerged as an important response to pollutants. PAH exposure can alter epigenetic mechanisms, including miRNAs [32]. miR-125b is a methylation modulator upregulated in cell cultures exposed to BaP and having inflammatory regulation functions [10]. Overexpression of miR-125b induces the expression of TNF-α, IL-6 and IL-1β in plasma from rheumatoid arthritis patients, showing a strong positive correlation between miR-125b and TNF-α [33]. Here, we showed that *TNF-α* and miR-125b expression were not increased in women from the high PAH exposure group. The difference between the work of Zhang et al. and our results could be attributable to other mechanisms that modulate the expression of TNF-α, including other miRNAs or another epigenetic mechanism, lower sample numbers, a different population and environment, as well as feedback mechanisms activated during inflammatory chronic process. In addition, we analyzed the expression of miR-155, which is a multifunctional microRNA. Recent data indicated that miR-155 has different expression profiles and plays a crucial role in various physiological and pathological processes such as hematopoietic lineage differentiation, immunity, inflammation, cancer and cardiovascular diseases [34]. It has been reported that in human alveolar macrophages, co-culture with miR-155 inhibitors increases TNF-α expression [35], showing similarity to our work, despite the expression of *TNF-α* and miR-155 not being statistically significant.

AhR ligands such as PAHs induce biological effects, including the induction of cytochrome P450 (CYP1B1) and other AhR-regulated genes. Interestingly, it has been reported that other endogenous molecules such as TNF-α have a modulator effect in both cytochrome and AhR expression [26]. In previous studies, it has been shown that AhR ligands induce CYP1B production in cell cultures, and when TNF-α was added, the expression of CYP1B1 was induced. Interestingly, cells co-stimulated with both BaP and TNF-α showed synergized effect, significantly increasing CYP1B1 expression [36,37]. In our results, this association was not found. Interestingly, the *AhR* expression recorded in response to PAHs is similar in several works; therefore, *AhR* is an important biomarker for PAH exposure.

## 5. Conclusions

This study primarily describes women’s exposure to PAHs in terms of urinary 1-OHP concentration, which was confirmed as a marker that can be used to classify PAH exposure. In fact, a stratification into two exposure groups has been proposed for the experimented levels of exposure, one at low levels of the 1-OHP indicator and one at high levels of 1-OHP. Even if these different levels were not explained by the type or intensity of the domestic source, the stratification was very useful to demonstrate that high levels of PAHs promote the expression of *AhR*, which is probably involved in pathway modulation in vivo, suggesting that cell signaling triggered by *AhR* may be implicated in several pathogenesis processes.

## Figures and Tables

**Table 1:** The 1-hydroxypyren urinary concentration of exposed women. The limit of detection (LOD) was 1 nmol/L (0.21825 µg/L).

Units	Mean	SD	PC25	PC50	PC75	PC95	Min	Max
μg/g creatinine	1.46	2.11	0.84	1.47	2.76	7.10	˂LOD	10.87
µg/L of urine	1.09	1.68	0.61	0.96	2.10	6.08	˂LOD	9.19

**Table 2:** Biochemical and anthropometric features analyzed by multiple *t*-tests of two-way ANOVA of urine samples exposed to PAHs.

	*p* Value	Mean of Group A	Mean of Group B
Age	0.109067	38.8261	46.3529
1-OHP	0.001267	0.848821	4.53468
Glucose	0.121731	92.8827	120.720
Total cholesterol	0.197449	165.233	181.997
HDL cholesterol	0.651043	46.2526	48.3669
LDL cholesterol	0.409356	94.2199	102.562
VLDL cholesterol	0.182865	25.3966	31.0680
Triglycerides	0.211349	126.983	153.343
Height	0.432416	1.46174	1.47686
Waist diameter	0.324131	85.4565	88.4571
Hip diameter	0.281592	97.9565	101.814
Weight	0.324085	55.5478	58.6314
BMI	0.508237	25.9577	26.7646

## Data Availability

Data supporting the reported results may be requested from the corresponding author.
